# Bonding performance and surface characterization of cold-bonded acetylated beech (*Fagus sylvatica* L.) laminated veneer lumber

**DOI:** 10.1038/s41598-023-48224-z

**Published:** 2024-02-19

**Authors:** Maik Slabohm, Holger Militz

**Affiliations:** Wood Biology and Wood Products, Burckhardt Institute, Georg-August University of Goettingen, Buesgenweg 4, 37077 Goettingen, Germany

**Keywords:** Structural materials, Composites

## Abstract

Acetylation of wood with acetic anhydride reduces the wood–moisture interaction, improves the dimensional stability and resistance against biodegradation. However, the adhesive bonding is affected by the modification, which is crucial to manufacture engineered wood products, such as laminated veneer lumber (LVL). In this study we report the bonding of 8-layered acetylated beech (*Fagus sylvatica* L.) LVL boards to 2-layered LVL beams. The beams were glued together at room temperature adding three common load-bearing construction adhesives: melamine–urea–formaldehyde (MUF), phenol–resorcinol–formaldehyde (PRF), and one-component polyurethane (PUR). The bonding performance was tested by assessing its dry and wet tensile shear strength (TSS) and wood failure percentage (WF). Also evaluated were the material's density and moisture content (MC). The surface was characterized prior to bonding by its pH, roughness, and contact angle (CA). The adhesive penetration was observed by fluorescence microscopy. Aside from MUF, applying PRF and PUR adhesives achieved good bonding performance on acetylated LVL and references. Acetylated LVL displayed a more hydrophobic behaviour, a higher pH, a somewhat smoother surface, and an increased density.

## Introduction

Beech (*Fagus sylvatica* L.) is one of the most important and highly available hardwood species in Europe, particularly in Germany. Its easy-to-impregnate sapwood sections^1^ make it appealing for wood modification, and its reasonably high mechanical properties compared to other European wood species means that it can be considered for wood construction. However, its dimensional stability in contact with moisture and its natural durability against biodegradation are limited^1–3^. Therefore, beech should not be exposed outdoors without additional protection, especially not in load-bearing structures.

One approach to reduce the wood–moisture interaction, and thereby improve these limitations, is the acetylation with acetic anhydride^4,5^. Acetylation of wood is well-known under the brand names ACCOYA^®^ for solid wood, and TRICOYA^®^ for wood fibers. Although softwood species such as radiata pine are frequently used for acetylation, hardwoods such as beech can also be modified^6–10^.

Thin veneers are appealing to wood modification in order to facilitate the modification of difficult to treat parts of wood, such as beech heartwood^1^. The chemical uptake into the inner parts of the veneers is additionally facilitated by lathe checks, which are a result of the peeling process of rotary-cut veneers. This allows an even and easy uptake of the chemical during modification. For example, so-called wet pockets^11^ are less likely to appear on thin veneers compared to thick solid wood.

To manufacture laminated veneer lumber (LVL) usually two bonding processes are applied: (1) primary bonding of veneers to boards and (2) an optional secondary bonding of the boards to thicker dimensions, for example to beams. Phenol–formaldehyde (PF) resin is typically used for the primary bonding. High temperatures are required to cure the resin. However, a temperature gradient from the outside to the core of the board limits the thickness during the primary bonding. To bypass the thickness limitation a secondary bonding at room temperature is often applied by using cold-curing adhesives such as melamine–urea–formaldehyde (MUF), phenol–resorcinol–formaldehyde (PRF), and one-component polyurethane (PUR). However, acetylated veneer has changed properties, which affects the bonding behaviour and later service life^12^.

Although there is previous research on the primary bonding of acetylated beech LVL, which involves bonding at high temperature^13–15^, less has been conducted on bonding acetylated LVL at room temperature. Most recent research has focused on the lengthwise connection of acetylated beech LVL using similar adhesives^16^. However, more research is needed to understand the surface bonding at room temperature.

In this study, the secondary bonding of acetylated beech LVL was compared to untreated reference LVL. Three adhesives for load-bearing construction (MUF, PRF, and PUR) were chosen to bond two primary bonded 8-layered LVL boards together to thicker dimension LVL. The bonding performance including adhesive penetration as well as various material and surface properties were evaluated.

## Material and methods

### Manufacturing of acetylated beech laminated veneer lumber

**Figure 1:**
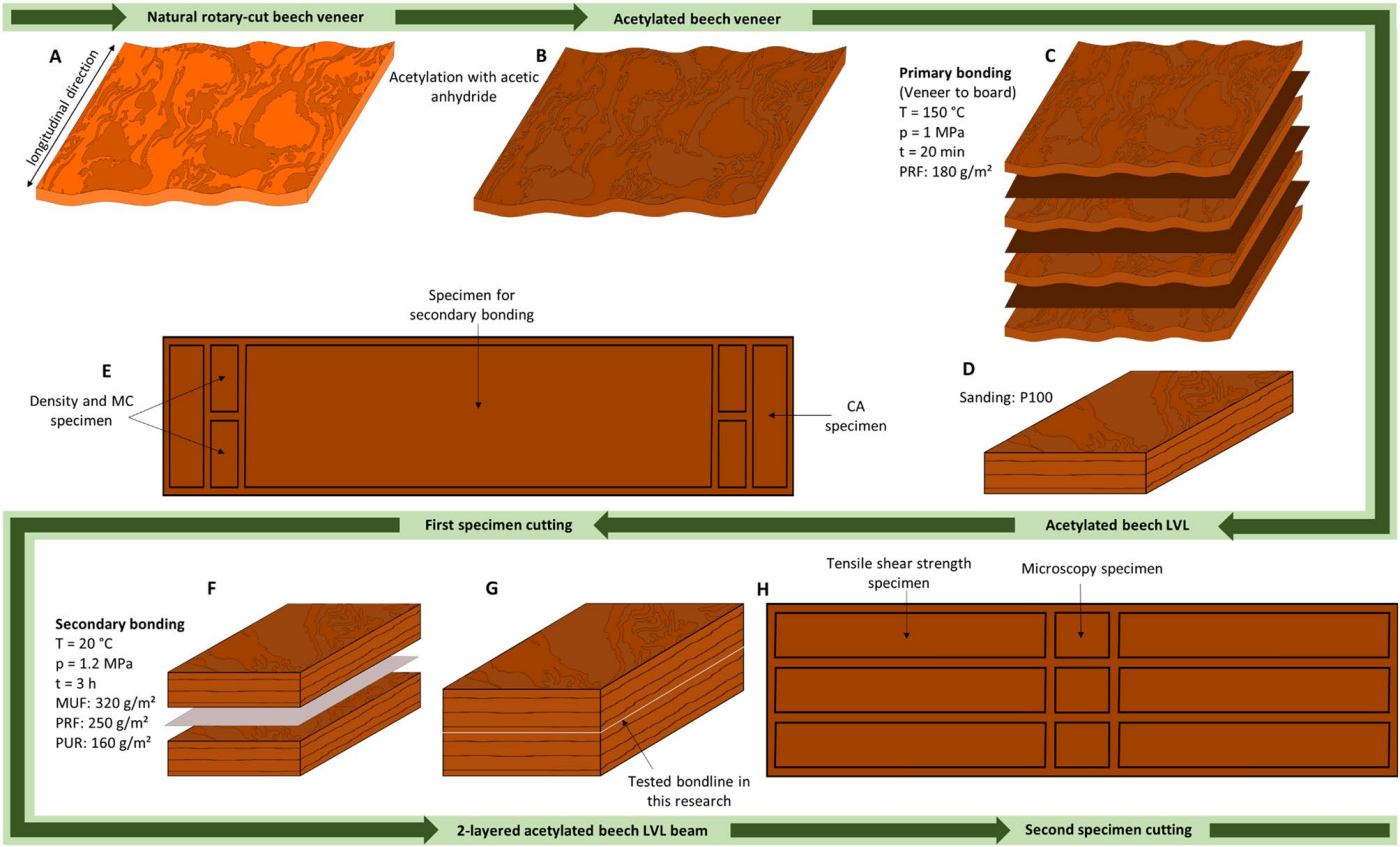
Schematic illustration of: (**A**) untreated veneer (**B**) acetylated veneer (**C**) primary bonding process (**D**) hot-bonded LVL board (**E**) top view of specimen allocation after first specimen cutting (**F**) secondary bonding process (**G**) cold-bonded LVL board (**H**) top view of specimen allocation after second specimen cutting; MUF = melamine-urea–formaldehyde, PF = phenol–formaldehyde, PRF = phenol-resorcinol–formaldehyde, PUR = 1-C Polyurethane, CA = contact angle, MC = moisture content.

Figure 1 provides an overview of manufacturing acetylated beech LVL, starting with the untreated veneer (A) and ending with the specimen preparation (H). A similar material for hot-bonding acetylated beech LVL as in previous studies was used^13^. Rotary-cut beech veneers (2200 × 1200 × 2.5 mm^3^) were acetylated with acetic anhydride at an industrial scale (Accsys Technologies in Arnhem, the Netherlands) to an weight percent gain (WPG) of approximately 24.4 (SD 0.5%).

Thereafter veneers were cut to 500 mm^2^ squares and hot-bonded using a two-component PRF resin. The liquid hardener Prefere 5839 (Dynea AS) was mixed with Prefere 4040 resin (Dynea AS) at a weight ratio of 20:100 to create the PRF resin. The PRF resin was chosen from Slabohm and Militz^14^ that indicated that it had the best bonding performance among other adhesive products. The adhesive was spread on 7 veneers of the 8-layered boards at an application rate of 180 g/m^2^. Afterwards, the boards were pressed at 150 °C for 20 min using a hydraulic press (LAP-40, Gottfried Joos GmbH & Co.KG). Untreated references were prepared using the same parameters. The LVL was stored in an unconditioned state for a number of weeks and protected from liquid water and direct sunlight.

Lamellas of 450 × 100 mm^2^ were cut out of the cooled LVL, planed to 10.5 mm thickness, and the surface was sanded one-sided (P100) to 10 mm thickness. This grid was chosen as it achieved good bonding of acetylated wood in another study^17^. No visible phenol adhesive from the primary bonding process remained on the clean surfaces after sanding. The boards were stored at 20 °C and 65% relative humidity (RH) for several weeks in a closed climate chamber for protection from dust, liquid water, and direct sunlight. The sanded surfaces were carefully protected as the specimen were cut in accordance to Fig. 1. With the use of compressed air, any possible remaining dust was removed.

Cold-bonding at room temperature (≈ 20 °C) was conducted using three commercially available adhesive products (MUF, PRF, and PUR) for load-bearing construction. Based on recommendations from manufacturers, these three products were selected. Adhesives were applied with a brush to suppliers’ requirements on one of the two sanded surfaces. For each specimen, two hot-bonded LVL lamellas of 350 × 100 × 10 mm^3^ were bonded together with 1.2 N/mm^2^ for 3 h using the same press as for hot-bonding, as mentioned above. To prevent the lamellas from shifting, pieces were fixed to a frame. There were four replicates for each material-adhesive combination (24 in total).

### Analysis of acetylated beech LVL

#### Density and moisture content

The oven-dry density was determined on the boards remainings (Fig. 1) by measuring length, width, thickness and mass after oven-drying (Eq. 1). The moisture content (MC) was then calculated according to Eq. (2).

1 $$\uprho = \frac{{\text{m}}}{l\times {\text{w}} \times {\text{t}} }{\times \,10^{5}} \left[ {{\text{kg/m}}^{{3}} } \right]$$

where ρ = density (oven-dry) [kg/m^3^], m = mass [g], l = length [mm], w = width [mm], t = thickness [mm]

2 $${\text{MC}} =\frac{ {{\text{m}}_{{{\text{20/65}}}} - {\text{m}}_{{{\text{dry}}}} } }{{\text{m}}_{{{\text{dry}}}}}\left[ \% \right]$$

where MC = moisture content [%], m_dry_ = oven-dry mass [g], m_20/65_ = mass conditioned at 20 °C and 65% RH [g]

#### Contact angle

The contact angle (CA) was measured using an automatic device (Mobile Surface Analyzer Flexible Liquid, Krüss GmbH, Hamburg, Germany) and measurements were conducted as in Slabohm et al.[16]. However, a droplet of 1 µl HPLC grade water was placed on the surface instead of adhesives. At an interval of 1 second, five measurements were made and analysed using the drop shape function Fitmethod Ellipse (Tang.-1). The CA was automatically calculated by the software ADVANCE (Krüss GmbH, Hamburg, Germany).

#### Surface pH measurement

The pH was measured using a flat-pH-electrode (PH CHECK F, Dostmann electronic GmbH). Measurements were conducted after 20 µl of demineralized water were precisely pipetted on the surface. The pH was taken at 20 ± 2 °C, after 30, 60, 180, and 300 s with the pH meter on the surface.

Alternative approaches, such as the measurement using water-soluble wood parts in an extraction^18^, were not chosen due as (1) the pH between surface and an extraction wood powder can differ^19^ and (2) additional adhesive of the primary bonded LVL is much likely to have also an impact on the pH. Furthermore, using only single veneer sheets for extraction were decided against, since the surface pH could vary as a result of the primary bonding at 150 °C. Therefore, solely the surface pH was measured^20,21^.

#### Laser-scanning-microscopy

The surface roughness was measured as described in Slabohm et al.^16^. A laser-scanning-microscope (LSM) (VK-X110, control unit: VK-X100, Keyence, Osaka, Japan) was utilized to compute various roughness parameters based on EN ISO 25178-1^22^.

#### Fluorescence microscopy

Fluorescent images were made using a Keyence microscope (BZX810 series, Osaka, Japan) with a 10 × objective lens (PlanFluor, NA 0.30, Ph1) and combinations of three channels (DAPI, GFP, TRITC) as well as various mono and colour settings were used. The lower and upper focus limits were set manually to make several images at an interval of several µm in Z-direction and the single images were merged together. Areas were measured at random.

Samples (Fig. 1, three per adhesive material combination) were stored in water for 3 h at 80 °C to facilitate the preparation. Cross sections were cut as smooth as possible using a using a manual sliding microtome. Optional staining was made with safranin or Astra blue (382291 and 382221, Dr. Hans-Jürgen Thorns Biologie-Bedarfs-Handel, Germany; Table 1).

**Table 1:** Staining of the LVL cross sections based on Lütkemeier^17^.

Material	Adhesive	Staining
Untreated	MUF, PRF, PUR	Dipping in 0.1% Safranin solution for 30 min, rinsing off with demineralized water, drying on a hot plate at 50 °C for 10 min, drying in an oven at 60 °C for 30 min
Acetylated	PUR	No staining
	MUF, PRF	Dipping in an Astra blue concentration for 5 min, rinsing off with demineralized water, drying on a hot plate at 60 °C for 10 min, drying in an oven at 60 °C for 30 min

#### Tensile shear strength and wood failure percentage

In total, 144 specimens (12 per adhesive-material-pretreatment combination) were tested on its tensile shear strength (TSS) and wood failure percentage (WF) based on EN 314-1^23^. Tests were carried out on the testing equipment Zwick/Roell (Ulm, Germany) with a maximum load cell of 10 kN and the textXpert^®^ III V3.5 (Zwick/Roell, Ulm, Germany) software.

Two pretreatments were chosen. Half of the specimens were tested in dry state (20 °C and 65% RH) and the other half were tested wet (after four hours boiling in water, 18 h drying at 60 °C, another four hours boiling in water and 2 h of cooling in water at room temperature).

The TSS was calculated according to the following Eq. (3) and the WF was determined in 5% steps. Specimens that were delaminated during the boiling-drying-boiling-cycle were rated with 0% TSS and 0% WF. The tested bonding area (A) for each specimen was measured in dry state (± 0.01 mm) before exposure to water.

3 $${\text{TSS}} = \frac{{\text{F}}_{\max }}{{\text{A}}}=\frac{{\text{F}}_{\max }}{{\text{a}} \times {\text{b}}\;}\left[ {{\text{N}}/{\text{mm}}^{{2}} } \right]$$

where TSS = tensile shear strength [N/mm^2^], F_max_ = maximum load [N], A = tested bonding area [mm^2^], a = length of bonding area [mm], b = width of bonding area [mm].

#### Statistical analysis

The software R^24^ was used for statistical computing and graphics. Boxplots were used to display the data set. Analysis of variance (ANOVA) was conducted to identify significant differences between the groups.

## Results and discussion

### Density and moisture content

**Figure 2:**
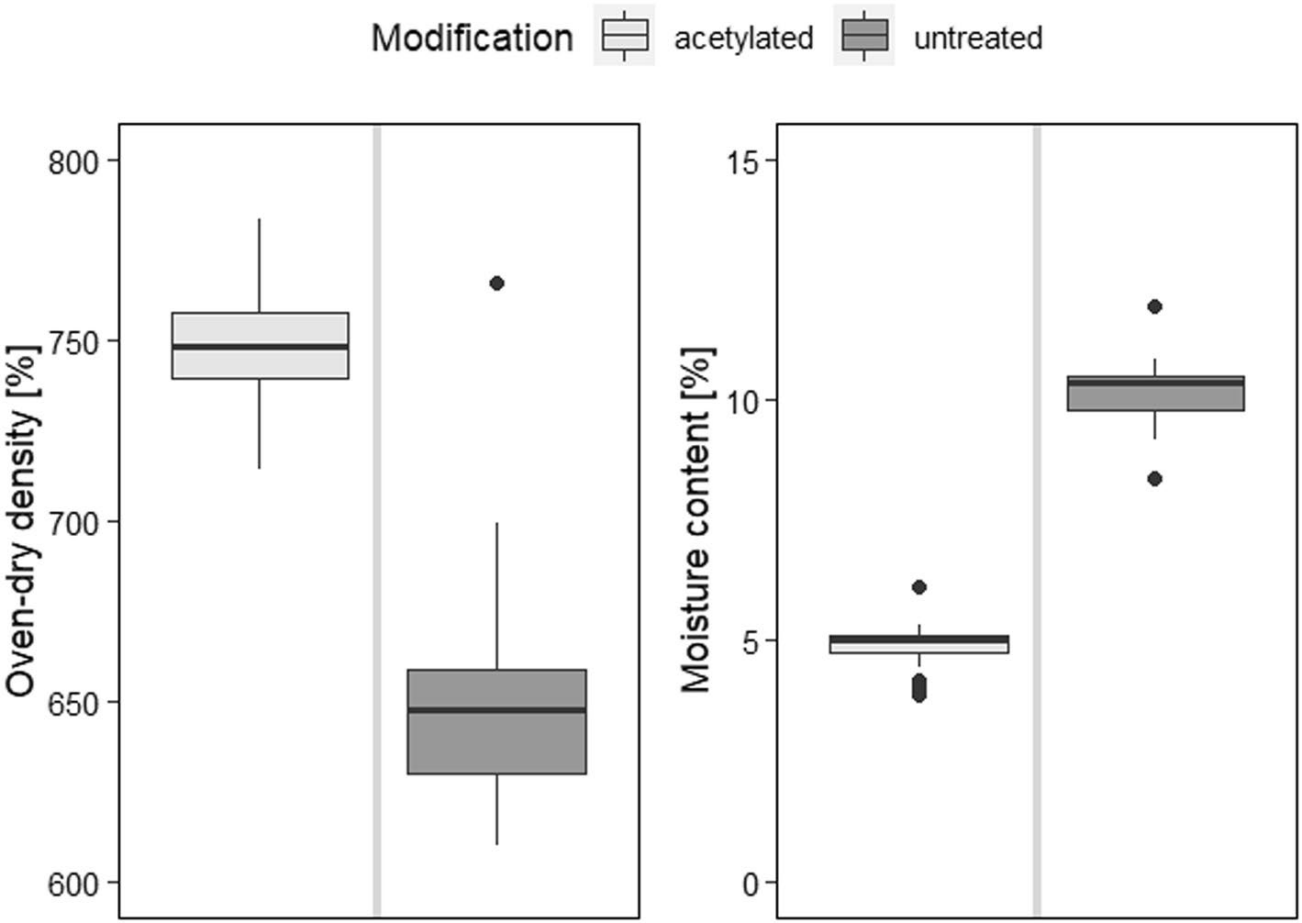
Oven-dry density and MC at 20 °C and 65% RH of acetylated beech LVL. Both, oven-dry density and MC, were found to be significantly (*p* < 2e−16) affected by modification (Supplementary Table 1).

As expected, the oven-dry density of the acetylated LVL was higher than the one of untreated references (Fig. 2). This can be explained by the additional acetyl groups that were added when the veneers were acetylated with acetic anhydride (WPG 24.4% (SD 0.5%)). There are a number of reasons why the oven-dry density of the acetylated LVL increased by only about 15% rather than by the WPG's percentage, when compared to the references. For instance, during primary bonding, when density is increased on the references, acetylated LVL displayed significantly smaller thickness reduction (densification)^13^. Another possible example is due to the changed mass-to-volume ratio as a result of bulking.

After conditioning at 20 °C and 65% RH, the MC on acetylated LVL is considerably lower when compared to the references (Fig. 2), which is in line with findings of many research studies^7,14, 15, 25^. Replacement of hydroxyl groups and cell wall bulking are the main contributors to the reduced MC^26–30^.

### Contact angle

**Figure 3:**
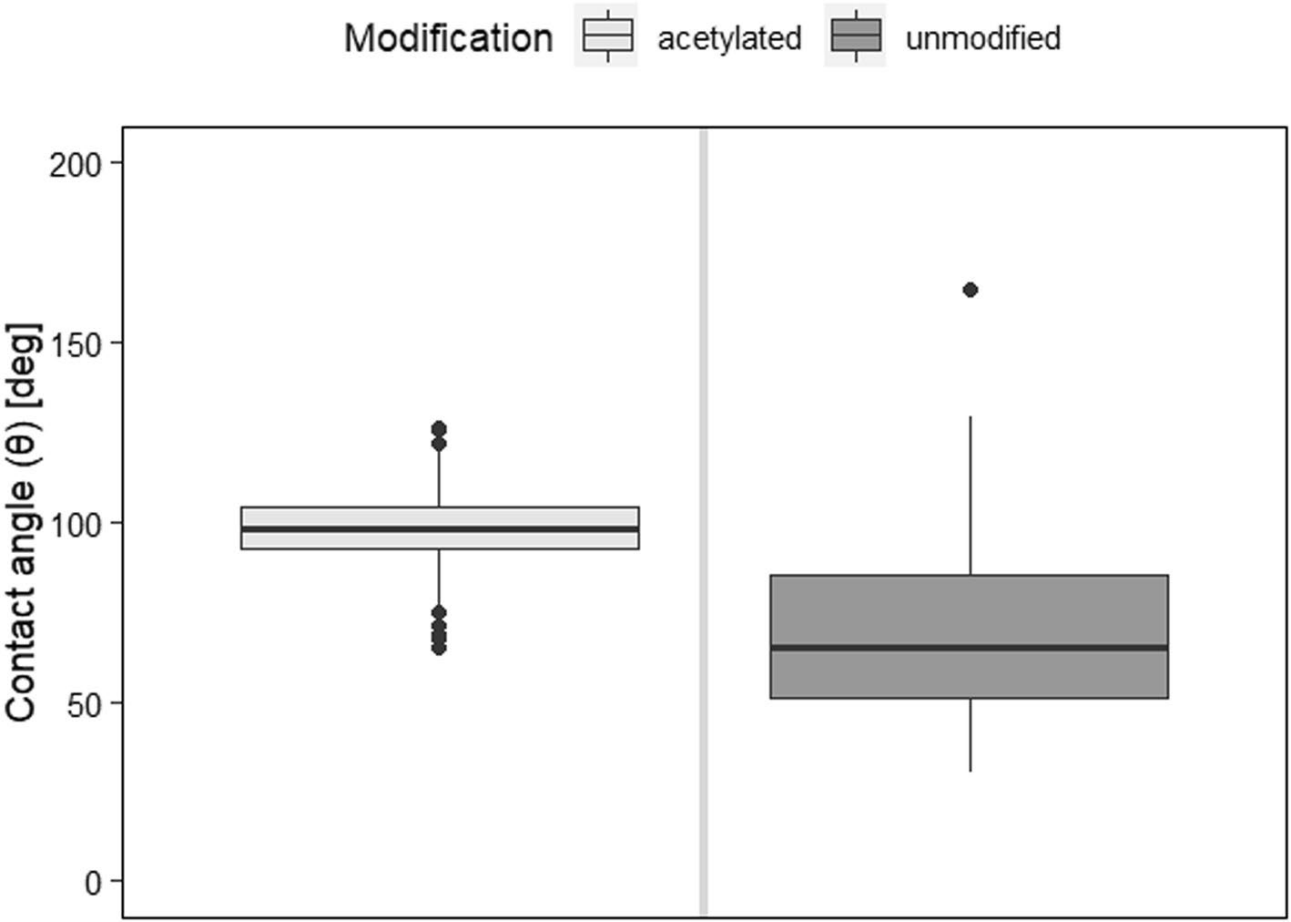
CA of sanded acetylated beech LVL after storing at 20 °C and 65%. CA was found to be significantly (*p* < 2e−16) affected by modification (Supplementary Table 1).

Acetylated beech LVL showed a higher degree of hydrophobicity than untreated specimens (Fig. 3). This is in line with several studies^31–34^ that demonstrate the hydrophobic behaviour of acetylated wood. The primary causes of this behaviour are the replacement of hydrophilic hydroxyl groups by the relatively hydrophobic ester groups, blocked pathways because of bulking, and changed extractives^34^. The more hydrophobic surface may have an impact on the adhesive penetration.

### Surface pH measurement

**Figure 4:**
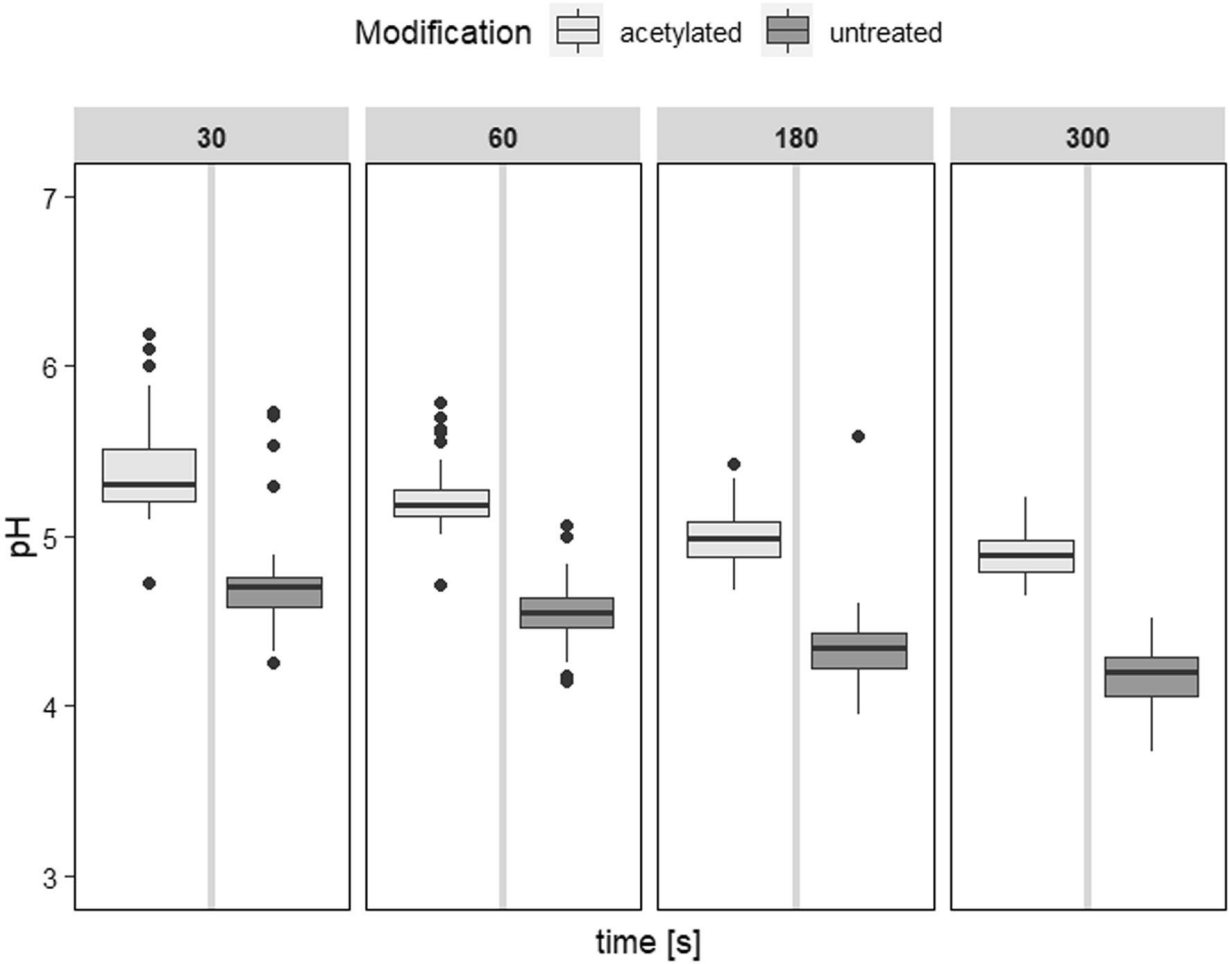
Surface pH after 30, 60, 180, and 300 s measurement time. pH was found to be significantly (*p* < 2e−16) affected by modification and time (Supplementary Table 1).

In this study, the concentration of hydrogen ions of the aqueous solution on acetylated LVL surface was lower as compared to untreated LVL (Fig. 4). Other studies, however, often found lower pH for acetylated specimens as compared to untreated references^35,36^. Though speculative, there are a number of potential causes for the higher pH of acetylated LVL.

Acetylation may decrease the amount of naturally existing acids and extractives. Acids, produced by the acetylation process, might have been washed out during and after the acetylation. This would reduce the wood-water interaction (see Figs. 2, 3), which would bring less water-soluble parts to the surface.

Another explanation could be that changes as a result of the primary bonding (up to 150 °C) occur in the wood. For instance, Sandermann et al. (1970) discovered significant amounts of volatile acids in untreated beech at temperatures as low as 125 °C, particularly after exposure at 150 °C. It's possible that acetylated wood forms fewer acids than wood that hasn't been treated.

According to literature, untreated beech had a pH on the surface of around 5^37,38^. There are marginal variations between untreated beech with discoloured red-heart parts those without, as well as between fresh and old surfaces^37^. The slightly lower pH in this study (4.57 ± 0.21 after 60 s) can be explained by subtle variations in the applied methods. For instance, because the flat electrode was already completely surrounded by water, only 20 µl rather than 150 µl^37^ of liquid were pipetted on the surface. As opposed to solid wood, the phenol adhesive between the veneers serves as a barrier to prevent extractives from penetrating from the core to the surface. Furthermore, 30, 60, 180, and 300 s were chosen as measurement times. As a result, the pH decreased with increasing measurement time. Finally, the heterogeneities of wood (for example, the number of extractives) have an impact on its pH.

### Roughness

**Figure 5:**
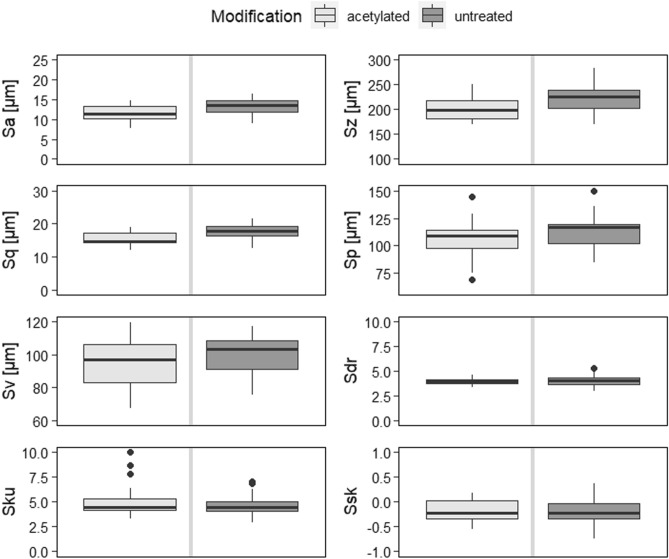
Surface roughness parameters: Sa (arithmetical mean surface height), Sz (maximum height), Sq (Root mean square height), Sp (maximum peak height), Sv (maximum pit height), Sdr (developed interfacial area ratio), Sku (kurtosis), Ssk (skewness). Sa (*p* < 0.0101), Sz (*p* < 0.0079), Sq (*p* < 0.00488), Sp (*p* < 0.0436), and Sv (*p* < 0.0463) were found to be significantly affected by modification (Supplementary table 1).

Various roughness parameters were calculated to investigate topographical variations between acetylated specimens and references (Fig. 5). The topography of acetylated LVL appears to have only minor differences compared to untreated LVL, which agrees with another study on freshly cut acetylated finger-joints^16^. Similar to the other study, the surface was slightly smoother (Sa, Sz, Sq, Sp_acetylated_ < Sa, Sz, Sq, Sp_untreated_).

### Microscopy

**Figure 6:**
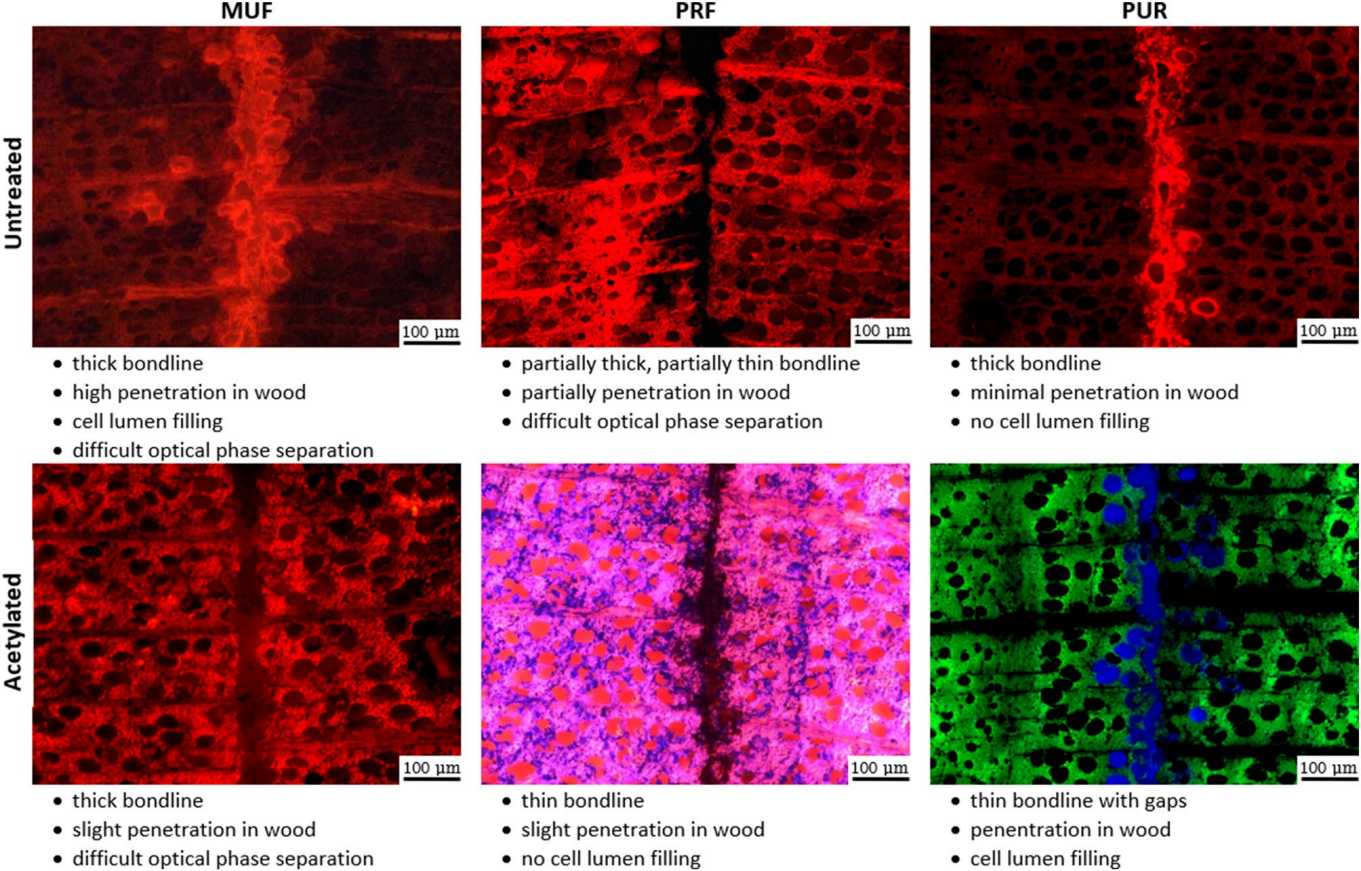
Bondline patterns of MUF, PRF, and PUR adhesives in acetylated and untreated beech veneer.

The bonding interfaces were visualized by fluorescence imaging of the specimen cross sections (Fig. 6). This allows an inside view on the adhesive penetration and distribution, which was qualitative described.

Not all adhesive-material combinations could achieve a clear optical separation. This issue might be solved by using other approaches^39–44^, for example other staining’s in combination with various settings.

Additionally, it is possible to add fluorescent dyes in the adhesive formulations of MUF and PRF. This was not included in the current study as it might alter the chemistry of the adhesive and affect bonding.

The adhesive penetration depends on many parameters such as open and closed assembly time, wood MC, pressure, wood species, applied adhesive amount, adhesive product and viscosity.

### Bonding Performance

**Figure 7:**
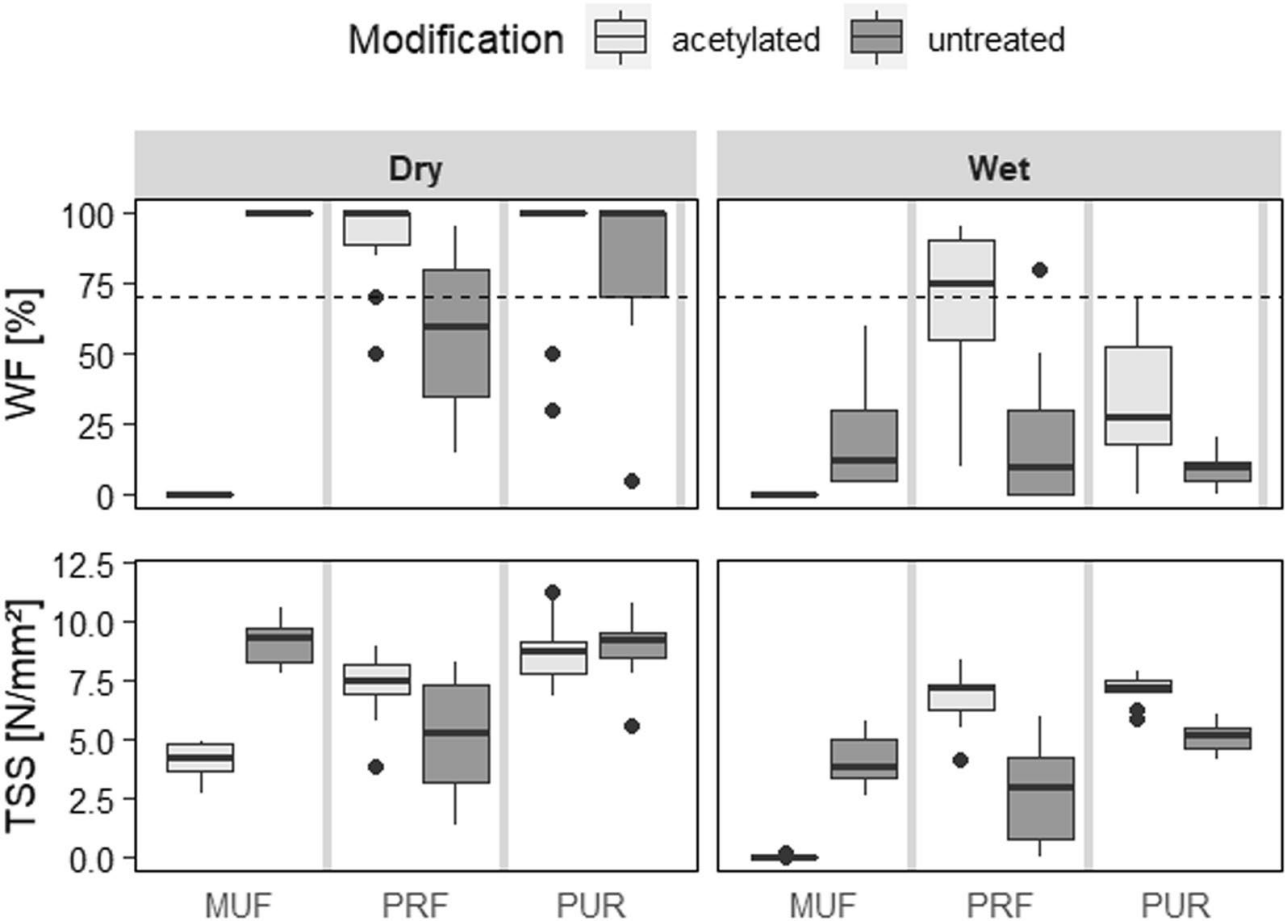
Bonding performance of 2-layered LVL beams bonded at room temperature using three different adhesives (MUF, PRF and PUR). The dotted line at 70% WF is based on EN 14374^48^, which specifies that LVL in load-bearing construction must have at least 70% WF. Since this method tests only the WF percentage and not the TSS, this research was based on EN 314-1, which is usually applied for plywood. On the other hand, the standard for LVL ^49^ refers to EN 314-1 for the bonding requirements. According to the specification of ^50^ the requirements for plywood in exterior use (in this case mean shear strength ≥ 1.0) were fulfilled for PRF and PUR for acetylated beech LVL. Supplementary Table 1 lists the results of ANOVA on WF and TSS.

To investigate if the bonding quality is influenced by the prior acetylation, TSS and WF percentage were evaluated (Fig. 7).

Acetylated specimens bonded with PUR and PRF showed best bonding performance. This is in line with other studies on bonding acetylated wood^14,16,17,36,45^. These acetylated specimens outperformed untreated references, particularly after the water treatment. This can be explained by two factors: (1) less shrinking and swelling in contact with moisture after acetylation^14^, which results in less stress inside the bonding; and (2) the shear strength of the acetylated wood is less affected in wet conditions than those of untreated references.

Acetylated specimens bonded with MUF showed lower dry TSS and consistently 0% WF, indicating low adhesion at the interface between wood and adhesive (Fig. 7).

Insufficient adhesion between the acetylated wood and the adhesive at the interface may have been brought on by the changed chemistry, such as the higher pH (Fig. 4) and the remaining acetic acid that was not washed out. That acetylated and untreated samples failed also in wet state can be explained by the low water stability of MUF^16,45–47^.

Surfaces were sanded several weeks before bonding, which may result in slight changes of the surface properties, even with additional protection from UV light and particles like saw dust. Higher bonding performance is expected by using freshly prepared surfaces (sanded, planed). Especially for the PRF adhesives, which was shown on freshly cut acetylated finger-joints^16^. On the other hand, veneer surfaces (rotary-cut) are generally not freshly prepared before bonding and achieve high bonding performance, for example untreated and acetylated beech LVL bonded with PRF adhesive^14^. In that study, even higher TSS and WF were achieved. One reason for this could be that the adhesive film in this study was thicker in comparison to the other. However, PRF is typically known for thorough bonding.

In general, bonding of wood is a highly complex process. Due to limited resources, one adhesive product (MUF, PRF, and PUR) was selected as a representative example of each adhesive type. The performance of other MUF, PRF, and PUR adhesives may perform slightly different. Additionally, the bonding might be enhanced by adjusting the process parameters, like the amount of applied adhesive, the open and closed assembly times, the pressing time, wood MC, and other variables.

## Conclusions

Bonding acetylated beech LVL was most effective using the selected PRF and PUR adhesive products. Even after a boiling cycle in water performed these bondings high. The data also indicated that acetylated specimens, bonded with the MUF adhesive product, had already poor bonding performance in dry state. This was likely due to changed chemistry after acetylation. For example, a higher pH was found on acetylated specimen compared to the references. Furthermore, a higher density, lower MC and higher CA on acetylated specimens were found but this had minor effects on adhesive penetration. The surface of acetylated LVL is slightly smoother as compared to reference LVL.

This work covers crucial insights on the secondary bonding of acetylated LVL. We further aim to manufacture larger acetylated beech LVL beams and thoroughly evaluate them on the bonding performance (for example delamination) in future research.

### Supplementary Information

Supplementary Information.

## Data Availability

The data used in this study is available from the corresponding author on reasonable request.
