# Supplementary material for: Bonding performance and surface characterization of cold-bonded acetylated beech (Fagus sylvatica L.) laminated veneer lumber
Source: Sci Rep. 2024 Feb 19;14:4083. doi: 10.1038/s41598-023-48224-z (PMC10876541; doi:10.1038/s41598-023-48224-z)
Supplement: Supplementary file 1 — Supplementary Information. [file 41598_2023_48224_MOESM1_ESM.docx]

# Supplementary Material

| Properties |  | Dependent variable | Influencing factors | p value | Significance level |
| --- | --- | --- | --- | --- | --- |
| Bonding performance |  | Tensile shear strength | Modification | 0.302 | ns |
|  |  |  | Adhesive | < 2e-16 | *** |
|  |  |  | Conditioning | < 2e-16 | *** |
|  |  |  | Modification:Adhesive | < 2e-16 | *** |
|  |  |  | Modification:Conditioning | 7.82e-05 | *** |
|  |  |  | Adhesive:Conditioning | 5.80e-07 | *** |
|  |  |  | Modification:Adhesive:Conditioning | 0.473 | ns |
|  |  | WoodFailure | Modification | 0.587946 | ns |
|  |  |  | Adhesive | 2.03e-11 | *** |
|  |  |  | Conditioning | < 2e-16 | *** |
|  |  |  | Modification:Adhesive | < 2e-16 | *** |
|  |  |  | Modification:Conditioning | 8.90e-08 | *** |
|  |  |  | Adhesive:Conditioning | 0.000173 | *** |
|  |  |  | Modification:Adhesive:Conditioning | 5.04e-05 | *** |
| Physical |  | Oven-dry density | Modification | <2e-16 | *** |
|  |  | Moisture content |  | <2e-16 | *** |
| Surface |  | Contact angle |  | <2e-16 | *** |
|  |  | Sa |  | 0.0101 | * |
|  |  | Sz |  | 0.0079 | ** |
|  |  | Sq |  | 0.00488 | ** |
|  |  | Sp |  | 0.0436 | * |
|  |  | Sv |  | 0.0463 | * |
|  |  | Sdr |  | 0.54 | ns |
|  |  | Sku |  | 0.465 | ns |
|  |  | Ssk |  | 0.211 | ns |
|  |  | pH | Modification | <2e-16 | *** |
|  |  |  | Time | <2e-16 | *** |
|  |  |  | Modification:Time | 0.397 | ns |

Supplementary table 1: Analysis of variance (ANOVA) ns: Not significant (p > 0.1); *: Significant (0.05 ≤ p ≤ 0.1); **: Significantly different (p ≤ 0.05); ***: Significantly different (p ≤ 0.01).
